# Species D Adenoviruses as Oncolytic Viral Vectors

**DOI:** 10.3390/v12121399

**Published:** 2020-12-06

**Authors:** Brianna L. Bullard, Brigette N. Corder, Eric A. Weaver

**Affiliations:** Nebraska Center for Virology, School of Biological Sciences, University of Nebraska, Lincoln, NE 68503, USA; bbullard@huskers.unl.edu (B.L.B.); brigette.corder@huskers.unl.edu (B.N.C.)

**Keywords:** adenovirus, oncolytic, hNIS, cancer, species D, alternative types

## Abstract

Oncolytic adenoviruses (Ad) have shown promising results in the therapeutic treatment of cancer. Ad type 5 (Ad5) is the most extensively utilized Ad type. However, several limitations exist to using Ad5 as an oncolytic virus, including high levels of anti-Ad5 neutralizing antibodies in the population, binding of the Ad5 hexon to blood coagulation factor X leading to liver sequestration and toxicity, and reduced expression of the primary receptor CAR on many tumors. Here, we use in vitro methods to explore the oncolytic potential of four alternative Ad types (Ad26, 28, 45, and 48) belonging to the species D Ad subgroup and developed replication-competent species D Ads expressing the human sodium iodide symporter protein (hNIS) for combination radiovirotherapy. We evaluated the species D Ad vectors transduction, replication, cytotoxicity, and gene expression in six different cancer cell lines. Species D Ads showed the greatest transduction and cytotoxic killing in the SKBR3 breast cancer cells, followed by 293, A549, and HepG2 cells, however the cytotoxicity was less than the wild type Ad5 virus. In contrast, species D Ads showed limited transduction and cytotoxicity in the Hela and SKOV3 cancer cell lines. These species D Ad vectors also successfully expressed the hNIS gene during infection leading to increased iodide uptake in multiple cancer cell lines. These results, the low seroprevalence of anti-species D antibodies, and the lack of binding to blood coagulation FX, support further exploration of species D Ads as alternative oncolytic adenoviruses against multiple types of cancer.

## 1. Introduction

Oncolytic virotherapy, or the use of replication-competent viruses with a lytic life cycle to kill cancer cells, has promising therapeutic anticancer potential [1]. Human adenovirus (Ad) is one of the most utilized oncolytic viruses for the treatment of cancer [2]. There are over 60+ known human Ad types which are divided into seven species (A-G) [3]. Ad type 5 (Ad5), a species C Ad virus, is the most commonly used type for oncolytic virotherapy and has shown success in both preclinical and clinical trials for treatment of multiple cancer types [1]. However, several limitations exist to using an Ad5 vector. First, an estimated 50–90% of the adult population is seropositive for pre-existing anti-Ad5 neutralizing antibodies (NAbs) [4,5,6]. These anti-Ad5 NAbs have previously been shown to limit the antitumor activity of the Ad5 type, especially during intravenous systemic delivery to treat metastatic lesions [7,8,9]. In addition, intravenous administration of Ad5 can lead to liver toxicity due to significant liver sequestration from Ad5 hexon binding to the blood coagulation factor X (FX) [10,11]. Finally, Ad5 uses the coxsackie-adenovirus receptor (CAR) which is often absent or downregulated in cancer cells [2,12,13,14,15]. Due to these limitations, researchers have begun exploring alternative Ad types for use as oncolytic viruses.

Species D Ads show many promising advantages that support their potential use in oncolytic virotherapy. We have specifically selected Ad26, 28, 45, and 48 from the species D Ad group because all four types have previously shown oncolytic activity against B-cell lymphomas [16,17]. Additionally, species D Ads show low seroprevalence rates of NAbs [5,18,19]. Studies in the USA have shown seropositivity to Ad26, 28, and 48 to be only 9%, ≈10%, and 3%, respectively [6,18,20]. In addition, species D Ads have been shown to utilize multiple receptors for cellular entry, such as CD46, CAR, sialic acid, glycans, and integrins, however, receptor usage remains controversial [16,21,22,23,24]. Finally, the hexon protein of species D Ads does not bind to blood coagulation FX, resulting in a lack of liver tropism after intravenous administration [11]. Biodistribution studies of Ad26 and Ad48 demonstrated no liver sequestration after intravenous delivery, in contrast to Ad5 [11]. The species D Ads low seroprevalence rate of NAbs, alternative receptor usage, and absence of liver toxicity warrants further investigation into species D Ads as an alternative oncolytic adenoviruse.

Importantly, the best cancer therapies are often multimodal. Therefore, we have also engineered these species D Ads to express the human sodium iodide symporter (hNIS) gene for virally directed radioisotope therapy, or radiovirotherapy. The hNIS is a membrane ion channel that transports iodide into cells [25]. Expression of hNIS by oncolytic viruses leads targeted killing of infected cancer cells by intracellular concentration of radioactive ^131^I [25,26]. Additionally, hNIS expression by oncolytic viruses allows for noninvasive nuclear imaging to monitor viral biodistribution and improve tumor targeting [26]. Combination treatment of oncolytic adenoviruses expressing hNIS and radioiodide therapy has shown increased anticancer activity as compared to the virus alone against multiple cancer types [25,27,28,29,30].

Here, we use in vitro methods to characterize the oncolytic potential of four species D Ads (Ad26, 28, 45, and 48) and develop four recombinant species D Ads expressing the hNIS gene for use as oncolytic and radiovirotherapy vectors. We investigate the species D Ads cellular transduction, transgene expression, cytotoxicity, and viral replication in six different cancer cell lines. Lastly, we assessed NaI uptake from expression of the hNIS gene.

## 2. Materials and Methods

### 2.1. Cell Culture

A549 (ATCC^®^ CCL-185™), HeLa (ATCC^®^ CCL-2™), SK-OV-3 [SKOV3] (ATCC^®^ HTB-77™), Hep G2 [HepG2] (ATCC^®^ HB-8065™), SK-BR-3 [SKBR3] (ATCC^®^ HTB-30™) were purchased from ATCC. Human kidney 293 cells were courtesy of Frank Graham (AdVec, Inc., Hamilton, CA, USA). All cell lines were maintained in DMEM supplemented with 10% FBS and 1% penicillin-streptomycin and incubated at 37 °C in a 5% CO_2_.

### 2.2. Recombinant Adenovirus Cloning

The wild-types viruses of Ad26, Ad28, Ad45, and Ad48 were obtained from ATCC. All four types were originally isolated from stool specimens [31]. Wild type Ad5 [ATCC VR-5] was obtained from ATCC and was originally isolated from adenoid tissue in 1953. The complete genomes of these Ads were cloned into a single low copy plasmid as previously described [32,33]. Either the GFP luciferase (GFP-Luc) reporter cassette or human sodium iodide symporter (hNIS) transgene replaced the adenoviral E3 gene from nucleotide position 26,530 to 30,690. Briefly, overlapping PCR was performed for upstream and downstream regions of the adenoviral E3 gene. The PCR primers incorporated PmeI restriction enzyme sites on both ends and a unique AscI site in the middle. The PmeI sites were used to ligate the PCR fragment into a shuttle plasmid containing a CMV promoter, the respective transgene, a polyA tail, and an FRT flanked zeocin gene. Each shuttle was linearized with AscI and cotransformed with 1 µg of the corresponding adenovirus clone for homologous recombination in BJ5183 electrocompetent cells. Recombinants were selected on LB containing Kanamycin and Zeocin and confirmed by restriction enzyme digest. Positive clones were transformed into XL-1 cells and midiprepped using a QIAGEN Plasmid Midi kit (Qiagen, Valencia, CA, USA). Recombinant replication-defective E1/E3 deleted adenovirus 5 expressing a GFP-Luc reporter cassette was created using the AdEasy Adenoviral Vector System (Agilent, Santa Clara, CA, USA) according to the manufacturer’s instructions.

### 2.3. Recombinant Adenovirus Virus Rescue, Purification, and Quantification

The recombinant adenovirus genomes were released from the plasmid backbone through digestions with PacI. Plasmids were buffer-exchanged with StrataPrep PCR purification kit (Agilent Technologies, Santa Clara, CA, USA) and transfected into 6-well plates of 293 cells using Polyfect Transfection reagent (Qiagen, Valencia, CA, USA). Cells were monitored for plaque formation and infected cells were subjected to three freeze–thaw cycles. Virus was amplified though sequential passage up to a Corning 10-cell stack flask (Millipore Sigma, Burlington, MA, USA). Virus was purified through two sequential CsCl gradients and a desalting column, Econo-Pac 10DG Desalting Columns (Bio-Rad, Hercules, CA, USA). The virus particle quantity was determined by OD_260_ nm using a Nanodrop Lite Spectrometer (Thermofisher, Waltham, MA, USA).

### 2.4. GFP Pictures and Flow Cytometry

To examine transduction, cell lines were seeded in a 12-well plate at 2 × 10^5^ cells/well. Cells were infected the next day with 500 virus particles/cell (vp/cell) of the indicated replication-competent species D Ad-GFP-Luc virus or replication-defective Ad5-GFP-Luc viruses. Transduction was evaluated at 24 h to eliminate potential differences from replication profiles. GFP expression was examined 24 h post-infection and cells were imaged with an EVOS FL Cell Imaging System (Thermo Fisher). GFP images were overlayed with DAPI images using Image J to visualize cell confluency. Transduction was quantified by harvesting and fixing cells 24 h post infection with Ad-GFP-Luc viruses and then counting GFP+ cells on a BD Accuri™ C6 Plus Flow Cytometer. FX binding was evaluated by preincubating Ad-GFP-Luc virus with media containing either buffer or physiological levels of blood coagulation factor X for 10 min at 37 °C (10 μg/mL; Haematologic Technologies; HCX-0050) before infecting confluent HepG2 cells at 500vp/cell and evaluating transduction by flow cytometry as described above.

### 2.5. MTT Assay

Cell lines were seeded in a 96-well assay plate (Corning, Corning, NY, USA) at 2 × 10^4^ cells/well and infected the next day with serial dilutions of the indicated wild type or Ad-hNIS virus ranging from 10^5^ to 10^0^ vp/cell. Five days post infection, 25 µL of MTT (4 mg/mL) was added to each well and incubated for 1 h at 37 °C to allow for formation of intracellular punctate precipitate. Media was then removed and 100 uL of acidified isopropanol solution (0.1 M HCl and 5% Triton X-100) was added before being read on a SpectraMax i3x Multi-Mode microplate reader (Molecular Devices) at OD_570_ nm. Percent cell viability was determined by comparison to uninfected wells of each cell line. The EC50 was determined by fitting a sigmoidal dose–response curve using the GraphPad Prism software.

### 2.6. qPCR Assay for Viral Replication

To examine Ad replication by qPCR, cell lines were seeded in a 12-well plate at 2 × 10^5^ cells/well and infected the next day with 500 vp/cell of the indicated Ad-hNIS virus. Total DNA was isolated from the cells at 24 and 72 h post-infection using the DNAeasy Blood and Tissue Kit (Qiagen Valencia, CA, USA). Real time PCR was performed using PowerUp SYBR Green Master Mix (Applied Biosystems) and run on the QuantStudio 3 Real-Time PCR System (Applied Biosystems) using the following conditions: 50 °C for 2 min, 95 °C for 2 min, 40 cycles of 95 °C for 15 s and 60 °C for 1 min, followed by a melt curve. Samples were run with Ad hexon primers Ad26/48 qPCR-F (5′-ACCGCCAGAGAACGCGCGAAGATGGCCACCC-3′) and Ad26/48 qPCR-R (5′-AGGCTGAAGTACGTGTCGGTGGCGCGGGC-3′) and normalized to levels of β-actin using primers ACTB gDNA qPCR-F (5′-GGCCTTGGAGTGTGTATTAAGT-3′) and ACTB gDNA qPCR-R (5′-GGACATGCAGAAAGTGCAAAG-3′). The results were compared to a standard curve created by dilution of pCR8 vector (Addgene) containing the Ad hexon fragment or β-actin gene and then results were converted to number of copies per cell.

### 2.7. NaI Uptake Assy

Sodium iodide symporter function was determined using a nonradioactive iodide uptake assay [34]. Cell lines were seeded in a 96-well assay plate (Corning, Corning, NY, USA) at 2 × 10^4^ cells/well and infected the next day with 500 vp/cell of the indicated Ad-hNIS or Ad-GFP-Luc virus. At 48 h post infection, media was replaced with 10 µM of NaI and incubated for 3 h to allow for NaI uptake. After 3 h, cells were washed once with DPBS and 100 uL of ddH20 was added to each well followed by 100 uL of 10.5 mM ammonium cerium (IV) and 100 uL of 24 mM sodium arsenite (II). After 30 min incubation, the plate was read on a SpectraMax i3x Multi-Mode microplate reader (Molecular Devices) at OD_420_ nm. NaI uptake was determined by comparison to a standard curve created by dilution of NaI.

### 2.8. Statistical Analysis

GraphPad Prism software was used to analyze all data. Experiments were repeated in triplicate and graphed data represent the means of data from all experimental replicates with error bars representing standard deviation. A *p*-value < 0.05 was considered statistically significant (* *p* < 0.05; ** *p* < 0.01; *** *p* < 0.001; **** *p* < 0.0001).

## 3. Results

### 3.1. Development of Replication Competent Species D Adenovirus

**Figure 1:**
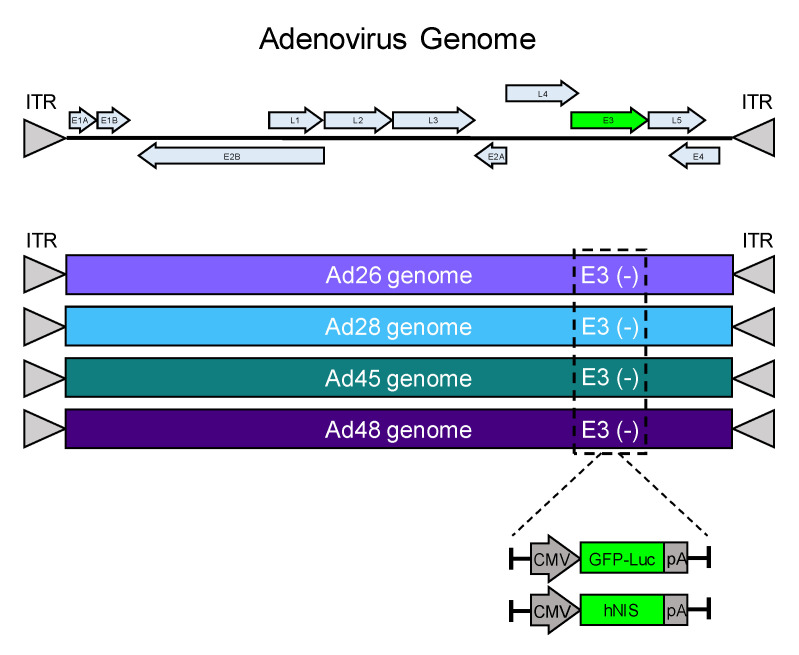
Schematic of recombinant species D oncolytic viruses. A schematic of an adenovirus genome is shown (top). The E3 genes from Ad26, Ad28, Ad45, and Ad48 were replaced with a GFP-Luciferase expression cassette (GFP-Luc) or a human sodium ion symporter (hNIS) gene. Each transgene included a CMV promoter and a flanking polyA tail.

We developed four replication competent species D adenovirus (Ad) vectors to evaluate their oncolytic potential in the treatment of cancer. The wild type genomes of Ad26, 28, 45, and 48 were each cloned into a single low copy expression plasmid as previously described [32,33]. The nonessential E3 gene for each adenovirus was deleted and replaced with a GFP-Luciferase expression cassette (GFP-Luc) to examine transduction and gene expression in cancer cell lines (Figure 1). Alternatively, the E3 gene was replaced by the human iodide symporter gene (hNIS) to create a recombinant oncolytic virus as a candidate for radiovirotherapy (Figure 1). The resulting eight unique recombinant viruses were evaluated for transduction, gene expression, cytotoxicity, and iodide uptake.

### 3.2. Viral Transduction and Transgene Expression

**Figure 2:**
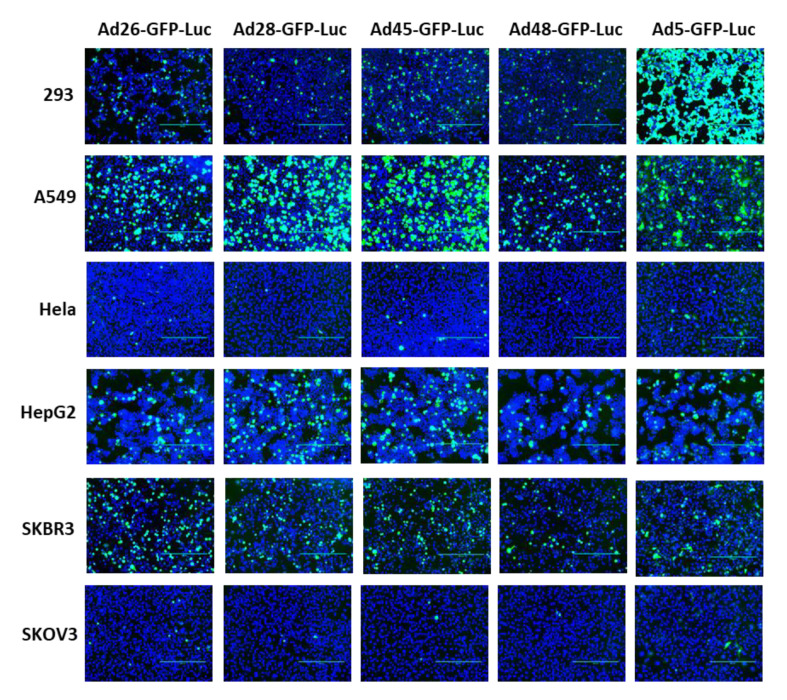
GFP gene expression of the recombinant species D viruses and Ad5 in six cancer cell lines. Each cell line was infected with 500 vp/cell of the indicated species D Ad-GFP-Luc virus and fluorescent images of GFP expression were taken at 24 h post-infection at 10× magnification. The GFP image was overlayed with the DAPI image using ImageJ to visualize cell confluency.

**Figure 3:**
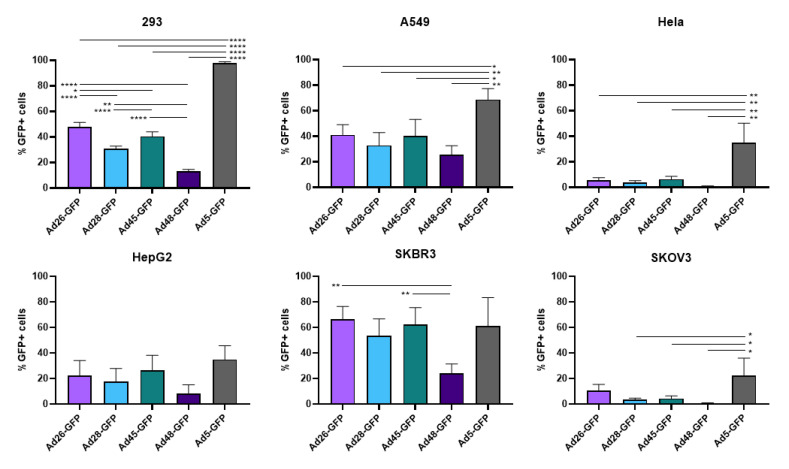
Viral transduction of the recombinant species D Ads or Ad5 GFP-Luc viruses. The transduction of the Ad-GFP-Luc virus was examined by infecting cells with the indicated viruses at 500vp/cell and determining GFP+ cells at 24 h post infection using flow cytometry. Data is presented as mean ± standard deviation (SD) from three independent experiments (* *p* < 0.05, ** *p* < 0.01, **** *p* < 0.0001; one-way ANOVA).

First, we examined the ability of these species D Ads to transduce various cancer cells and compared this transduction to Ad5 as a benchmark. Our study utilized six cell lines from multiple different tissues to characterize the potential oncolytic activity against multiple cancer types (Table 1). We used the recombinant Ad-GFP-Luc viruses to easily visualize and quantitate viral transduction. Cells were infected with the indicated Ad-GFP-Luc virus and evaluated for GFP expression after 24 h using fluorescence microscopy (Figure 2). Transduction was quantified by measuring GFP+ cells 24 h post infection using flow cytometry (Figure 3). The four species D vectors showed the strongest transduction in SKBR3 breast cancer cells, followed by A549, 293, and then HepG2 cells. Species D Ads showed equivalent transduction levels to Ad5 in both the SKBR3 cells and HepG2 cells. While species D Ads successfully transduced both 293 and A549 cells, this transduction was less than the Ad5 virus. The species D vectors showed the weakest GFP expression and transduction in Hela and SKOV3 cell lines, however the Ad5 virus also showed the weakest transduction in these two cells lines. These results indicate that the species D Ads are best at transducing SKBR3 cells, followed by A549, 293, and HepG2 cells, while transduction of Hela and SKOV3 cells is more limited.

### 3.3. Binding to Blood Coagulation Factor X

**Figure 4:**
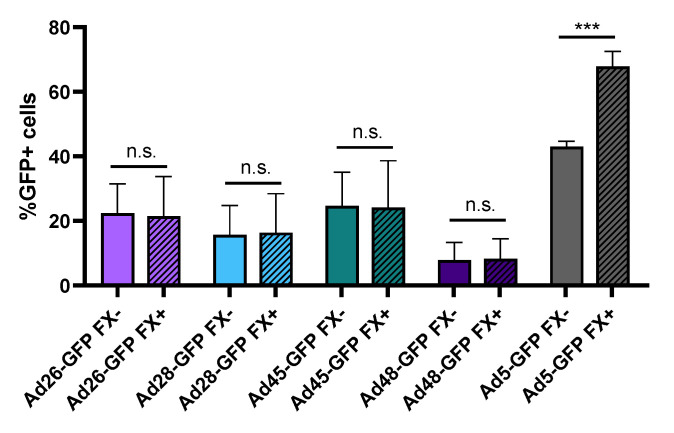
Hepatocyte transduction in the presence of blood coagulation factor X. The effect of FX binding was measured by changes in hepatocyte transduction. Each recombinant adenovirus was preincubated with or without physiological levels of FX prior to infection of HepG2 cells at 500 vp/cell. Transduction was evaluated 24 h later by determining GFP+ cells using flow cytometry. Data is presented as mean ± standard deviation (SD) from three independent experiments (n.s.—not significant, *** *p* < 0.001; Student’s *t*-test).

One major limitation to using Ad5 as an oncolytic virus is the strong binding of the Ad5 capsid hexon protein to blood coagulation factor X (FX). This Ad5-FX complex increases hepatocyte transduction through interaction with heparin sulfate proteoglycan on the hepatocytes and results in liver sequestration and toxicity [11,35]. In contrast, previous studies have shown that species D Ads do not bind FX or demonstrate liver sequestration [10,11]. To support this finding, we preincubated Ad-GFP-Luc expressing viruses with media containing either buffer or physiological levels of FX for 10 min and then infected HepG2 hepatocytes. After 24 h, we evaluated virus transduction by determining GFP+ cells using flow cytometry (Figure 4). Our results confirm that presence of FX in the media increases Ad5 hepatocyte transduction but has no significant effect on the transduction of the species D viruses. This supports previous studies demonstrating that, unlike Ad5, species D Ads do not bind to blood coagulation FX. This lack of binding to FX reduces liver sequestration and toxicity following intravenous administration and therefore has important implication for systemic administration of oncolytic viruses [10,11].

### 3.4. EC50 of Wild Type Viruses in Cancer Cell Lines

**Figure 5:**
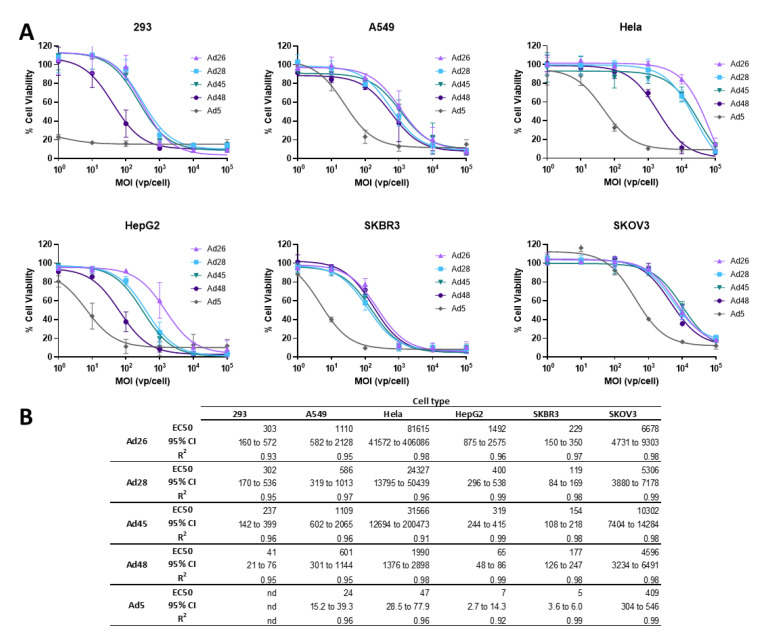
Oncolytic activity of the wild type species D Ads and wild type Ad5. (**A**) The half maximal effective concentration (EC50) of the wild type viruses were determined by infecting cells with serial dilutions of the indicated virus and evaluating cell death 5 days post infection by an MTT assay. Cell viability was determined by comparison to uninfected cell controls. Data is presented as mean ± standard deviation (SD) from three independent experiments. (**B**) EC50s, 95% confidence intervals, and R^2^ values are shown in the table.

Next we evaluated the ability of these species D Ads to result in cellular lysis and cell death of cancer cells [1]. To evaluate the cytotoxicity of these species D viruses for use as an oncolytic virus, we first determined the half maximal effective concentration (EC50) of the wild type viruses in each cancer cell line and compared that to the EC50 of the wild type Ad5 virus as a benchmark. Cell lines were infected with serial dilutions of the indicated wild type virus and an MTT assay was performed at 5 days post infection (Figure 5). Cell viability is evaluated at 5 days post infection to allow for a round of viral replication and spread within each cell line [36].

Cytotoxic killing was detected in all cell lines for all species D vectors, however the cytotoxicity was less than that of the wild type Ad5 virus (Figure 5A). Nevertheless, the species D Ads still showed high cytotoxicity in the SKBR3 and 293 cell lines, with EC50s between 119–229vp/cell in SKBR3 cells and 41-303vp/cell in 293 cells (Figure 5B). Species D viruses also demonstrated high-to-moderate cytotoxicity in the HepG2 and A549 cell lines. However, the Hela and SKOV3 cell lines only showed cytotoxicity during high MOI conditions of species D Ads, likely due to the lower transduction ability seen in these cell lines. Interestingly, the Ad48 virus showed the highest cytotoxicity out of the four species D Ads in 293, HepG2, and Hela cells while all four species D viruses showed similar EC50s in the A549, SKBR3, and SKOV3 cell lines. These results show that, although species D Ads have reduced cytotoxic killing as compared to Ad5, these viruses still have promising cytotoxic activity, particularly against SKBR3, 293, HepG2, and A549 cancer cell lines.

### 3.5. EC50 of the Recombinant Ad-hNIS Viruses in Cancer Cell Lines

**Figure 6:**
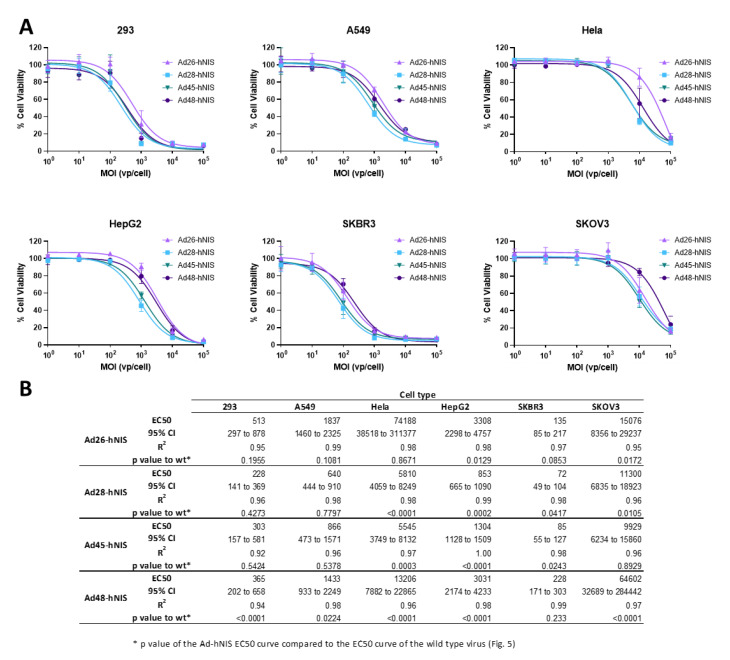
Oncolytic activity of each recombinant species D Ad-hNIS virus. (**A**) The half maximal effective concentration (EC50) of the four species D Ad-hNIS viruses was determined by infecting cells with serial dilutions of the indicated virus and evaluating cell death 5 days post infection by an MTT assay. Cell viability was determined by comparison to uninfected cell controls. Data is presented as mean ± standard deviation (SD) from three independent experiments. (**B**) Table of EC50s, 95% confidence intervals, R^2^ values, and *p* value of the Ad-hNIS EC50 curve compared to the wildtype EC50 curve.

After evaluating the cytotoxic activity of the wild type viruses, we next determined the EC50 of the recombinant species D viruses expressing hNIS using the same methods (Figure 6). These recombinant viruses showed a similar pattern of cytotoxicity to their wild type counterpart, with the highest cytotoxicity observed in the SKBR3 and 293 cell lines, followed by A549 and HepG2 cells, and weak cell killing in the Hela and SKOV3 cell lines. Importantly, in the SKBR3 cell line, the Ad-hNIS E3 deleted viruses showed similar or better cytotoxic killing as compared to the wildtype counterparts (Figure 6B). In addition, in 293 and A549 cells, there was no significant difference between the EC50 curves for wild type and Ad-hNIS E3 deleted viruses for Ad26, 28, and 45, but there was a significant difference for Ad48 (Figure 6B). The Ad48 type showed the most pronounced reduction in cytotoxicity between Ad-hNIS E3 deleted and wild type virus in all the cell types except SKBR3 cells. These results indicate that there is similar cytotoxicity between the Ad-hNIS E3 deleted and wild type viruses in SKBR3, 293, and A549 cells.

### 3.6. gDNA Replication Kinetics in Each Cancer Cell Line

**Figure 7:**
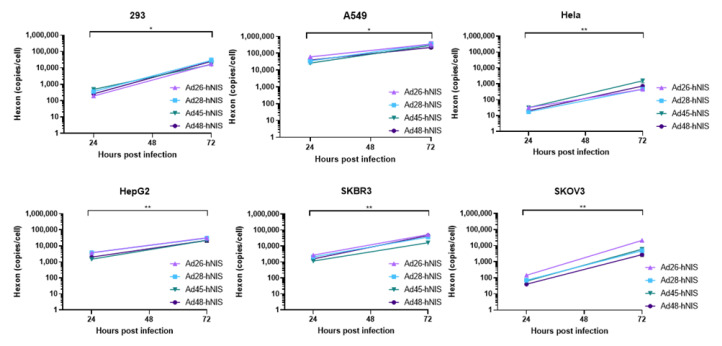
Replication kinetics of the recombinant species D Ad-hNIS viruses in the cancer cell lines. The gDNA replication kinetics of the four species D Ad-hNIS viruses were examined in each cancer cell line using qPCR for the Ad hexon gene. Cells were infected with 500vp/cell of the indicated Ad-hNIS and DNA was isolated at 24 and 72 h post infection. Hexon copies are displayed per cell as determined by the β-actin gene. Real-time qPCR was performed in triplicate and presented as mean ± standard deviation (SD) (* *p* < 0.05, ** *p* < 0.01; two-tailed *t*-test).

Next, we evaluated the replication capability of the species D Ad-hNIS viruses in each cancer cell line by using quantitative polymerase chain reaction (qPCR) to examine genomic amplification over time. DNA was isolated from Ad-hNIS infected cells at 24 and 72 h post infection for detection of Ad gDNA by qPCR. All four species D Ad vectors showed significant genomic amplification between the 24 and 72 h timepoints (Figure 7), indicating successful virus replication in each cancer cell line. At 72 h post-infection, the highest gDNA copies/cell occurred in the 293, A549, HepG2, and SKBR3 cells, while lower gDNA copies/cell were observed in Hela and SKOV3 cells, likely due to lower initial transduction in these cell lines. However, significant gDNA amplification still occurred in Hela and SKOV3 cells between 24 and 72 h post infection, indicating that replication can still occur. These results suggest that species D Ad vectors can replicate their gDNA in each of the cancer cell lines.

### 3.7. Increased NaI Uptake after Ad-hNIS Infection

**Figure 8:**
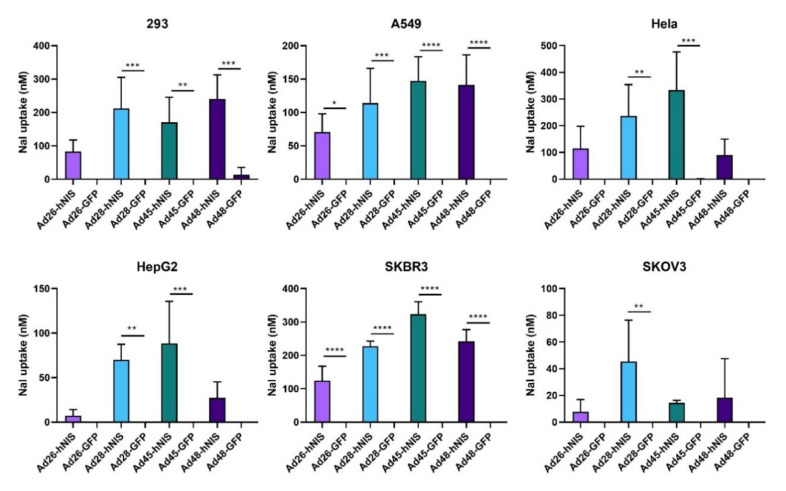
Sodium iodide uptake after infection with the recombinant species D Ad-hNIS viruses. The iodide uptake after infection by the species D Ad-hNIS viruses was evaluated by infecting cell with the indicated viruses at 500 vp/cell and performing a nonradioactive iodide uptake assay after 48 h post infection. NaI uptake after Ad-hNIS infection was compared to NaI uptake after Ad-GFP-Luc infection to control for Ad infection and replication. Data is presented as mean ± standard deviation (SD) from three independent experiments (* *p* < 0.05, ** *p* < 0.01, *** *p* < 0.001, **** *p* < 0.0001; one-way ANOVA).

Lastly, we determined the iodide uptake after infection with the Ad-hNIS vectors to confirm successful expression of a functional hNIS protein. A nonradioactive iodide uptake assay was performed 48 h post infection with an Ad-hNIS or Ad-GFP-Luc control virus [34]. All four species D vectors showed significant sodium iodide (NaI) uptake in SKBR3 and A549 cells (Figure 8). Infection of 293 cells resulted in significant NaI uptake in all vectors except Ad26-hNIS. Ad28-hNIS and Ad45-hNIS showed significant NaI uptake in both HepG2 and Hela cells. However, all vectors showed relatively weak NaI uptake in the SKOV3 cell line. These results show that expression of the hNIS by species D vectors leads to successful iodide uptake in multiple cancer cell lines.

## 4. Discussion

Human Ad5 is the most extensively used oncolytic Ad type in the treatment of cancer. However, the high levels of anti-Ad5 neutralizing antibodies in the population, significant liver sequestration and toxicity from Ad5 hexon binding to blood coagulation FX, and reduced expression of the primary receptor CAR on many tumors limits the anticancer efficacy of this type. The need for alternative Ad types in oncolytic treatments promoted us to investigate species D Ads, which show low seroprevalence rates in the population and a lack of species D Ad hexon binding to blood coagulation FX. Here, we investigated the oncolytic potential of four species D Ads (Ad26, 28, 45, and 45) using in vitro methods and develop a replication-competent species D Ads expressing hNIS for combination radiovirotherapy.

First, we evaluated transduction efficiency in six different cancer cell lines and found that all species D Ads were able to transduce SKBR3, 293, A549, and HepG2, but only weakly transduce the Hela and SKOV3 cell lines. Notably, the species D Ads transduced SKBR3 and HepG2 cells at high levels similar levels to the Ad5 virus. Importantly, the primary cellular receptor usage for these species D Ads has not been fully determined and remains controversial in the literature. For example, some studies have suggested that Ad26 uses CD46 as a cellular receptor [5,16,22,23] while other studies challenge the utilization of CD46 and instead suggest CAR [37,38], αvβ3 integrin [39], scavenger receptor SR-A6 [40], sialic acid-bearing glycans [41], or another uncharacterized receptor as the primary receptor. Receptor usage for Ad48 also remains controversial [5,16,23,38,42] while receptor usage for Ad28 and Ad45 has not been explored in depth [20,23]. Differences in receptor expression by each cell line, especially uncharacterized receptors, could explain the lower transduction levels seen in the Hela and SKOV3 cell lines.

Importantly, unlike the Ad5 virus, preincubation of species D Ads with blood coagulation FX did not result in increased transduction of hepatocytes. This data supports the previous finding that the hexon capsid protein of species D Ads does not bind to blood coagulation FX and therefore does not exhibit liver sequestration and toxicity [10,11]. Biodistribution studies of Ad26 and Ad48 after intravenous administration have previously been performed and demonstrated no liver sequestration, however, further in vivo studies are needed to support the potential systemic administration of species D Ads in the treatment of cancer. The inclusion of the luciferase transgene in our recombinant Ad viruses facilitates future live imaging biodistribution studies.

Species D Ads have previously been explored for their increased transduction and oncolytic potential against B cell cancers [16,17], and here we show their potential against solid cancers. Although the species D Ads showed lower cytotoxicity as compared to Ad5 in each cell line, the species D Ads still showed promising cytotoxicity which may be effective against diverse cancer tissues. The species D Ads showed the highest cytotoxicity in the SKBR3 cell line, followed by 293, A549, and HepG2 cells, which have cellular origins from the breast, kidney, lungs, and liver, respectively. In addition, the cytotoxicity EC50 curves were similar between the wild type viruses and the recombinant hNIS expressing E3 deleted viruses in SKBR3, 293, and A549 cells. Further studies are needed to explore the oncolytic activity of these species D Ads in other cancer cell lines originating from these tissues. In addition, future studies to examine transduction and replication in healthy primary human cells are needed to examine the potential for off-target toxicity. If toxicity is observed, the recombinant species D Ads could undergo further genetic engineering to make replication cancer cell-specific in order to limit off target toxicity. Although these species D Ad vectors are currently replication competent, they can be further engineered to conditionally replicate in cancer cells. One strategy is to mutate the E1A or E1B Ad genes. In healthy cells, these Ad genes interfere with the cellular tumor suppressors retinoblastoma protein (pRB) and p53, respectively, to allow for successful viral replication [14]. Therefore, mutation of these Ad genes would limit replication to cells with defective tumor-suppressor pathways, such as most cancer cells [2]. Furthermore, the vectors could be modified with cancer- or tissue-specific promoters to limit off-target effects during radiovirotherapy [14].

Finally, expression of the hNIS gene by the oncolytic species D Ads resulted in cellular iodide uptake and concentration, with the strongest concentration observed in the SKBR3 cancer cell line. Expression of the hNIS gene by these species D Ads could enhance the anticancer potential by improving tumor targeting through noninvasive nuclear imaging or by direct cell killing from intracellular concentration of radioactive ^131^I [26]. However, further in vivo biodistribution and efficacy studies are needed to support the radiovirotherapeutic potential of these species D Ads.

Here, we evaluated oncolytic potential of four species D Ad types (Ad26, 28, 45, and 45) in vitro. We found that the species D Ad vectors showed the most promising cytotoxic activity and gene expression in the SKBR3 breast cancer cells, followed by 293, A549, and HepG2 cell lines, with limited activity in Hela and SKOV3 cells. Combined with radiovirotherapy, these species D vectors could provide a dual mechanism for targeting several carcinomas, especially in cancers that are refractory to standard treatment. Studies evaluating oncolytic efficacy, biodistribution, and radiovirotherapy treatments in vivo would further support the therapeutic activity of these species D viruses. Excitingly, the initial in vitro results of this study, paired with previous evidence of low seroprevalence and lack of liver toxicity, supports the continued development and characterization of these species D Ads as alternative oncolytic adenoviruses against multiple types of cancer.

## 5. Conclusions

Here, we evaluated the oncolytic potential of four species D Ad types (Ad26, 28, 45, and 45). Although the cytotoxicity of these species D Ads was less than Ad5, these species D Ad vectors still showed promising transduction, cytotoxic activity, and gene expression in the SKBRS, 293, A549, and HepG2 cell lines. The initial in vitro results of this study, paired with previous evidence of low seroprevalence and lack of liver toxicity, supports the continued development and characterization of these species D Ads as alternative oncolytic adenoviruses against multiple types of cancer.

## Figures and Tables

**Table 1:** Cancer cell line origins for the cell lines used in this research article.

Cell Line	Cell Origin
293	Embryonic kidney
A549	Lung carcinoma
Hela	Cervical carcinoma
HepG2	Hepatocellular carcinoma
SKBR3	Breast carcinoma
SKOV3	Ovarian carcinoma
